# Implications of Hepatitis E Virus in Blood Transfusions, Hemodialysis, and Solid Organ Transplants

**DOI:** 10.3390/medicina56050206

**Published:** 2020-04-25

**Authors:** Essam M. Janahi, Saba F. D. Parkar, Sakina Mustafa, Zaki M. Eisa

**Affiliations:** 1 Department of Biology, College of Science, University of Bahrain, Sakhir 32038, Bahrain; sabaparkar@outlook.com (S.F.D.P.); smustafa@uob.edu.bh (S.M.); 2 The National Center for Disease Prevention and Control, Jazan 82722-2476, Saudi Arabia; Zaki2006@gmail.com

**Keywords:** hepatitis E virus, seroprevalence, HEV genotype, chronic hepatitis, transfusion transmission, blood donors, prevention, chronic kidney disease, hemodialysis and organ transplantation

## Abstract

Hepatitis E Virus (HEV) is emerging as the primary cause of acute viral hepatitis in humans. The virus is commonly transmitted by the fecal–oral route via contaminated water in endemic regions or through the consumption of inadequately cooked swine products or game meats in industrialized regions. HEV genotypes 1 and 2 are predominantly associated with waterborne transmission in developing countries, whereas *HEV3* and *HEV4* are mainly zoonotically transmitted in industrialized countries. Seroprevalence in populations determined by detecting anti-HEV antibodies and serum HEV RNA is commonly used to analyze the presence of HEV. Although HEV RNA-based detection is now standardized, there is a lack of agreement between the assaying methods used for gathering seroprevalence data. Since 2004, HEV has been considered as a transmissible infectious agent through blood transfusion. Recent seroprevalence studies in European countries indicate an underestimated risk for blood transfusion and hence warrant testing the blood supply. HEV infection is usually self-limiting and spontaneously cleared. However, in about 60% of recipients of solid organ transplants, HEV progresses to chronic hepatitis. Immunosuppressive drugs such as tacrolimus are a major cause of chronic hepatitis and reducing its dosage results in viral clearance in about 30% of patients. In hemodialysis patients, the parenteral route is implicated as an important mechanism of transmission. In this review, we explore the clinical and epidemiological characteristics of various HEV genotypes in blood donors, hemodialysis patients, and transplant recipients.

## 1. Introduction

In the last decade, a change of the epidemiology of liver disease has been observed with an increasing trend towards non-viral etiology. However, virus-related hepatitis remains the main cause of liver disease worldwide [1,2]. The most common causes of viral hepatitis are the five hepatotropic viruses, hepatitis A virus (HAV), hepatitis B virus (HBV), hepatitis C virus (HCV), hepatitis D virus (HDV), and hepatitis E virus (HEV). In 1980, the bulk of hepatitis cases in India were attributed to a hitherto unrecognized pathogen [3]. Three years later, an experimental infection of an HAV-immune volunteer from the research team led to the discovery of HEV. After ingestion of pooled stool extracts from presumed hepatitis patients, he too developed hepatitis. The volunteer’s serum did not contain anti-HAV IgM and HBV. Spherical 27–30 nm viruses were detected by immuno-electron microscopy; these were inoculated in cynomolgus monkeys, where they successfully caused hepatitis and elicited an antibody response [4]. Identification and sequencing of the virus followed; the disease was thereafter known as hepatitis E, with HEV as the causative pathogen [5]. This organism is most appropriately allocated the letter “E”, which could stand for “enteric”, “epidemic”, or “endemic”, all of which are features ascribed to the epidemiology of HEV [6]. HEV is possibly the most prevalent cause of acute viral hepatitis around the globe [7]. HEV has a small particle with a positive-sense RNA genome (≈7.2 kb) [8]. HEV particles are now considered as “quasi-enveloped particles”, existing as non-enveloped virions in the feces and bile of infected individuals, while cellular membrane-bounded particles are found within the bloodstream [9,10,11]. According to a recent proposal, HEV is classified as a member of the *Hepeviridae* family with two genera. The first genus, *Orthohepevirus* has four species, *Orthohepevirus A*, with eight genotypes including those infectious to humans, and *Orthohepevirus B, C,* and *D,* which are not infectious to human. The other genus *Piscihepevirus* contains one non-human-infecting species [12]. All human-infecting HEV genotypes belong to species *Orthohepevirus A*. Genotypes 1 and 2 infect humans exclusively, while *HEV3* and *HEV4* infect a wide range of animals in addition to humans. Genotype 7, which was isolated from camels, is also infectious to humans, while genotypes 5 and 6 infect wild boars and are not known to infect humans [12]. All the genotypes of mammalian HEV are represented by one serotype [13]. There are many known reservoirs of HEV, with pigs as the most important reservoir. Other species of animals also serve as HEV reservoirs [13]. HEV infections can cause fulminant hepatic failure, and, with genotypes 1 and 2, a devastatingly high maternal mortality is seen primarily in the second and third trimesters [14,15,16]. HEV is not only responsible for acute sporadic or epidemic viral hepatitis but has been recently shown to be associated with chronic hepatitis as well [17].

## 2. General Epidemiology

HEV has emerged as the most common etiological agent of adult acute viral hepatitis in Central and Southeast Asia and is implicated as the next biggest cause, after HBV, in the Middle East and North Africa [18]. Genotypes of mammalian HEV (*HEV1–4*) display not only modes of transmission and clinical characteristics but also have geographical niches. Hepatitis E is typically reported to show one of the two marked epidemiological patterns; it either causes large outbreaks or sporadic cases [7,19]. Yearly sporadic cases and occasional outbreaks involving numerous cases may occur intermittently in developing countries [7]. In outbreaks, the clinical attack rate of about 1 in 2 is seen and some studies report males to be twice as likely to be affected [20,21]. *HEV1* is primarily reported in Asia and Africa, whereas *HEV2* is found in Mexico and Africa. *HEV3* is predominantly reported in Europe, USA and other industrialized western countries while *HEV4* is found in South East Asia, mainly in Japan, China, and Taiwan [22]. *HEV1* and *HEV2* cause self-limiting hepatitis in young adults, though there is an increased fatality in pregnant females and immunocompromised patients. *HEV3* and *HEV4* can cause symptomatic hepatitis in middle-aged and older individuals [23]. The first identified epidemic of hepatitis E was reported in Delhi, India, in 1955–1956 [24]. Since then, a number of sporadic, as well as large, outbreaks are reported each year [25,26].

*HEV1* is implicated in many outbreaks of viral hepatitis in India [27,28,29], Pakistan [30], Bangladesh [29], Egypt [31,32], and Venezuela [33]. Sewage contamination of potable water supplies near water supply stations and broken pipelines traversing sewer utilities may be responsible for the large outbreaks [24]. There are five subtypes (*1a–1e*) of *HEV1*; subtype *1c* is generally found in China, India, and Kyrgyzstan, while subtypes *1d* and *1e* have been only reported in Africa [19]. In India and Bangladesh, the same subtype *1a* is seen to be the cause of acute hepatitis [6,29,34]. *HEV1* is the predominant genotype seen in India, and certain subtypes such as *1c* may be implicated as causative of fulminant hepatitis [27].

Genotype 2 sequences of HEV (*HEV2*) are grouped into two subtypes, subtype *2a* from Mexico and subtype *2b* found mainly in several African countries such as Nigeria and Chad [22,35]. The presence of *HEV3* in the United States, Japan, Korea, Netherlands, and Mexico is well documented. Locally acquired infections in Europe and North America are mostly due to *HEV3,* and it is the cause of most autochthonous infections [36]. The subtypes *3a–3j* are found in developed countries [22,37]. Infected meats of game, swine, and boars are implicated in the zoonotic transmission of *HEV3* [38]. Swine is a potential animal reservoir of *HEV3* and *HEV4* with HEV prevalence of 7–15% in pigs, according to studies in the Netherlands and Belgium [37]. A study carried out for the detection of HEV RNA in pork liver and meat products in Dutch markets has shown that liver, liverwurst, and liver pate had the highest level of *HEV3* RNA [39]. In Japan, pig liver has been implicated as an important risk factor because 90% of patients who consume inadequately cooked or grilled pig liver two to eight weeks before have an onset of hepatitis symptoms [40]. In France, pig liver sausage displayed the presence of *HEV3* RNA in 59% of the samples bought in supermarkets; significant genetic similarities were observed between these sequences and those isolated from patients who ate the sausages [41]. Furthermore, HEV RNA was found in swine sera, and swine anti-HEV antibodies were also found in around 81% of the Mexican pigs [42]. In England, indigenous cases were due to *HEV3* strains, which were distinctive and strongly linked to *HEV3* strains from British pigs [36]. Another study in England showed HEV RNA detection in 3% of pig liver samples and 10% of sausage samples [43]. In the United States, among the pig samples collected from different slaughterhouses, 6.3% of the samples were viremic for HEV RNA, with less than 100 to 10^6^ copies/mL range of viral loads [44]. In Korea, HEV RNA was extracted from human sera gathered from various regions, and it was found to be closely related to three Korean swine isolates, with 92.9–99.2% nucleotide sequence homology [45].

Phylogenetic analysis of worldwide HEV sequences suggests that the point of origin for *HEV3* lies in the western hemisphere from where it was transmitted to Japan, Korea, and Taiwan, while *HEV4* is native to Asia. Additionally, *HEV3* and *HEV4* sequences from humans and animals were well conserved, suggesting a common persistent source or reservoir [46]. It has been shown that *HEV4* infections cause more severe human clinical outcomes, and this may have significant consequences for public health. Although most cases of autochthonous hepatitis E are caused by *HEV3*, a study in France showed the presence of *HEV4* was strongly correlated to swine HEV [47]. It was initially thought that HEV is responsible for exceedingly few cases of hepatitis in the developed countries [18]. In fact, researchers calculated the incidence of HEV infection at 0.7% in the USA [48]. However, a large number of symptomatic hepatitis E infection cases are “overlooked” as there are no currently available United States Food and Drug Administration (FDA)-approved diagnostic assays in humans [49]. *HEV3* and *HEV4* are zoonotic, and transmission is food-borne from animals [50].

## 3. Diagnosis of HEV

Molecular and serological methodologies are used to detect HEV in stool or serum [51]. HEV is usually diagnosed by serological assays relying on anti-HEV IgM and/or IgG. Immunochromatographic assays can also be used [52]. Alternatively, the detection of HEV RNA in blood and other bodily fluids is used for diagnosis [20]. 

HEV serological diagnosis is generally based on enzyme immunoassays (EIA). Immunodiagnostic target for these assays may be synthetic genes encoding several immunodominant antigenic epitopes from HEV structural proteins [53]. IgM anti-HEV is increasingly being used in routine diagnosis due to increased availability in many countries and improved reliability [54]. The four genotypes of the HEV show the same serotype [18] because HEV, even of different origins, stimulates antibody production by use of conserved epitopes of the virus.

HEV RNA is frequently used as a marker for acute hepatitis E infection. Reverse transcription–polymerase chain reaction (RT–PCR) assays are available that can detect genetically divergent HEV strains. Molecular studies and phylogenetic analysis have shown that the swine HEV strains are clustered in the same genotype as human HEV strains [55]. In order to standardize nucleic acid amplification based assays, World Health Organization (WHO) established 6329/10 as the standard for HEV RNA, with a unit of 250,000 IU/mL [56]. This step was taken in a bid to enhance inter-laboratory results for the detection and quantification of HEV RNA. A single-step betaine-free RT-loop-mediated isothermal amplification (LAMP) assay with a 100-fold greater sensitivity than ordinary RT–PCR was developed to diagnosis HEV rapidly [57]. An immunochromatographic test was developed by the use of recombinant protein EP2.1, derived from open reading frame (*ORF2*), and the monoclonal antibody 4B2 to detect HEV IgM antibodies in patients with acute hepatitis. The test is rapid, highly specific, and with 96.7% accuracy [52].

Acute infection by HEV is characterized from studies of experimentally infected animals by anti-HEV IgM antibodies, followed by the anti-HEV IgG after a few days [51,58,59,60]. Anti-HEV IgM titres decline rapidly during early period of recovery while the IgG antibodies have been detected for periods longer than 14 years and provide protection against subsequent infections [61]. Researchers speculate that it is not feasible to detect the incidence of sporadic hepatitis E accurately and with certainty unless HEV-RNA, IgM, and rising IgG levels are all used as markers of HEV-caused acute hepatitis [54].

## 4. HEV in Blood Transfusion

Blood can be transfused as “whole blood”, collected and stored in an anticoagulant. However, in order to satisfy the needs of more patients, blood can be divided into its parts, including red cell concentrate, fresh frozen plasma, cryoprecipitate and platelet concentrate. Some blood products have a short shelf life compared to others. The unavoidable delay between donation and the identification of a viraemic donor means that by the time the recall of components is initiated, the short shelf-life components may already be transfused. At best, longer shelf-life red cell and frozen components can be recalled [62]. The virus is viable in the blood and blood products for a significant time. Colson et al. (2007) reported a case where the recipient developed hepatitis six weeks after transfusion of blood products. The virus remained viable in the blood for four weeks after collection [63]. 

### 4.1. Seroprevalence in Europe

In developed European countries, HEV ranges between 0.4% and 3% [63]. The first report of HEV transmission in UK from a blood component was described retrospectively in 2006. Twenty-one days after blood donation, the donor developed jaundice. The recipient’s blood showed *HEV3* genotype, which was identical to the genotype from the implicated donor [64]. Further research on 880 plasma mini pools (42,000 individual donors) from London showed that HEV RNA exists in 0.7% of the samples. Additionally, 73% of HEV RNA negative samples were HEV IgG reactive, indicating a high rate of asymptomatic infection [65]. Anti-HEV seroprevalence of about 13% occurs in the general English population and it is estimated that almost 60,000 cases occur per year [66]. Hewitt et al. suggested that *HEV3* infections are present in the English blood donors but with low seroprevalence of 0.04% [62].

In France, the prevalence of anti-HEV antibodies in blood banks was 3.2%, which is similar to that of other industrialized countries. However, the HEV IgG prevalence among blood donors from Midi-Pyrénées, a province in southern France, is 52.5%, the maximum rate described in developed countries, implying that HEV is hyperendemic to Midi-Pyrénées [67]. Seroprevalence rates among Dutch blood donors is 20.6%; these high rates of HEV are perplexing investigators as Denmark is not located near an endemic region nor does it have explained risk factors responsible for the illness [68]. However, rates are considerably lower in Switzerland at 4.9%. The low seroprevalence may be due to differences in sensitivity of assay method used and the stringent laws regulating animal and meat imports in the country [69].

Industrialized countries have an HEV seroprevalences of 0.3–53% but these differences can be in part be attributed to detection assays dissimilarities [70]. Comparisons between studies face a fundamental challenge due to differences in the investigated populations and in the accuracy of the detection assays. Researchers in the United Kingdom found that two commercial assays showed dramatically different results of 3.6% and 16.2% while estimating the anti-HEV seroprevalence from 500 blood donors; it is believed that HEV prevalences lower than 5% in developed countries are typically obtained by the use of insensitive assays [71]. Other reports also state this issue, as shown by the substantial differences in diagnostic sensitivities of three tested commercial assays in Germany and South Korea [70,71,72]. In fact, Mansuy et al. (2008) carried out two studies in the same region of France and found that seroprevalence estimates were 3.1 times higher when the assay method was changed [67,73]. Furthermore, HEV enzyme immunosorbent assays (ELISA) based on *HEV1* and *HEV2* present in endemic settings might not fully represent *HEV3* and *HEV4* present in industrialized countries [69].

### 4.2. Seroprevalence in Asia and Africa

HEV infection is quite common in Africa, India, and Southeast Asia, where it is described as the main cause of acute hepatitis. Bangladesh shows a high seroprevalence of 22.5% in a random population study of 1134 specimens [74]. Unhygienic living conditions coupled with poor sanitation lead to higher anti-HEV seroprevalence among blood donors. The main source of outbreaks in endemic regions is sewage drainage into water supplies [24]. A study in India reported seroprevalence of 4.78%, which is comparable to Switzerland rather than other developing countries [75]. However, the study reports seroprevalence based on assays using anti-HEV IgM and care should be used while comparing seroprevalences as different researchers use varied assays.

The prevalence of HEV infection in the war-stricken Khuzestan Province, Iran, was 11.5% among blood donors. Interestingly, all patients were free of human immunodeficiency virus (HIV), HBV, and HCV [76]. High HEV seroprevalence in this region might be due to river pollution from surrounding factories and also unsanitary living conditions caused by war. Egypt is endemic to HEV as the incidence of anti-HEV IgG was 45.2% among blood donors [77]. An earlier study showed that children living in homes without a clean supply of water had a higher chance of hepatitis E infection [78]. Serum samples from 178 blood donors in Burkina Faso from 2010–2012 showed 19.1% seroprevalence of anti-HEV IgG [79]. The prevalence of HEV in Saudi Arabia was 16.9% [80]. It is widely recognized that HEV is transmitted through swine products. However, non-swine meats are also implicated in the transmission of HEV. Despite the prohibition of swine in Islamic countries, rates of HEV seroprevalence are quite high. Thus, studies on HEV from other animals’ meats used for human consumption may assist us to know better the epidemiology of HEV.

Blood transfusion represents a high-risk factor in contracting HEV in China. Chinese blood donors were 32.60% anti-HEV IgG seropositive and HEV viremia was 0.07% [81]. In the Philippines, 85 blood bags were assessed for the existence of HEV IgG and IgM antibodies; the prevalence of acute HEV infection using the IgM anti-HEV was 2.4% [82]. In Japan, 7.1% of a large cohort of blood donors (6700 individuals), with high liver alanine aminotransferase (ALT), tested positive for anti-HEV IgG [83].

### 4.3. Seroprevalence in America and Argentina

In Paraná, Brazil, anti-HEV IgG was found in 2.3% of the blood donors. The seroprevalence rate is comparable to those of blood donors in developed countries as well as to other regions in Brazil, such as 3.0% in São Paulo, 2% in Salvador and 4.3% in Rio de Janeiro [84]. The HEV seroprevalence rate in Argentine blood donors ranges from 1% to 2% and their phylogenetic examination indicates the discovery of a new genotype of HEV [85]. 

The seroprevalence of anti-HEV among blood donors in northern California is 1.2–1.4%. The data is consistent with that from prior seroprevalence studies (1–5%) among blood donors in the United States [86]. Seroprevalence studies from eight US states indicate that 18% and 17% of normal blood donor samples were anti-HEV positive when tested with human HEV antigen and swine HEV antigen, respectively. Despite the low numbers reported, it must be remembered that most infections are “missed” due to lack of FDA-approved human diagnostic assays [49].

## 5. HEV among Hemodialysis Patients

Several studies have shown possible associations of parenteral route of HEV infection in patients with chronic hemodialysis (CHD). It is has been shown that patients undergoing CHD are at higher risk for contracting parenterally transmitted microorganisms [87]. CHD patients showed a higher incidence of HEV infection, further corroborating evidence towards a blood-borne spread of the infection; hence, the probability of HEV transmission through the parenteral path in endemic areas was suggested [88]. Anti-HEV antibodies are more frequently found in blood transfusion recipients compared to the controls, which further supports the theory that parenteral route is another important route of HEV transmission [89]. Higher rates of seroprevalence in CHD patients compared to a control group would support this as well [90]. In fact, seroprevalence investigations in developed countries show a significantly higher rate of anti HEV antibodies in CHD patients as compared to the general population [91].

Conversely, there are reports where the rates of anti-HEV antibody were lower than in the CHD patients compared to the general population. Fabrizi et al. (1997) reported a 3% seroprevalence of anti-HEV in Italy [92]. Another study in Sweden indicates that HEV is not a hospital-acquired by patients on hemodialysis and there is no proven association between chronic hemodialysis and an enhanced probability of contracting HEV infection [93]. Researchers in Iran showed that seroprevalence of HEV was 2.2% lower in CHD patients in comparison to the general population. Nonetheless, the CHD sample was not from the same region of Iran and could potentially be the cause of the conflicting result [88]. Another study from the same country, showed the incidence of anti-HEV IgG was 10.6% in CHD patients [94]. However, a recent paper from the same region, with a case–control study where a control population was matched from the same geographical region, showed almost 20% lower rates of HEV antibody than CHD patients [95].

Psichogiou et al. (1996) carried out a multicenter hemodialysis cohort study. Although their study reported higher rates of anti-HEV antibodies in CHD patients when compared to a reference of normal population, they determined that this difference was not significant. CHD patients were not at higher risk of being infected with HEV and there was no substantial difference in HEV seroprevalence of patients with a history of blood transfusions compared to a healthy reference population [96]. These results were further corroborated by studies which failed to find a substantial correlation between anti-HEV IgG positivity and CHD in Turkey (20.6% HEV seropositivity in hemodialysis (HD) patients) and Greece (4.8% HEV seropositivity in HD patients) [91]. None of these studies could identify a substantial correlation between HEV infection and either renal disease, history of hepatitis B/C, or history of elevated transaminases.

Data concerning anti-HEV antibody prevalence among CHD patients are insufficient and provide contrasting results. Different HEV prevalences in the healthy population in different geographical locations, the selection criteria of the different studies, and the different transmission routes of HEV could be key in understanding these contrasting results. Additionally, regional differences, genetic variations of HEV and false-positive outcomes from other infections could explain the different results [95]. More retrospective studies with large number of HD patients or case control studies, where patients are matched with a control, are needed. Lastly, standardization of assay methods used will help pinpoint whether anti-HEV IgG seropositivity is acquired during hemodialysis.

## 6. HEV in Solid Organ Transplant Recipients

Organ-transplant recipients are susceptible to chronic active hepatitis due to HEV infection that may lead to a prompt progression to cirrhosis [97]. Chronic hepatitis is usually confirmed by persistently elevated aminotransferase levels, presence of HEV RNA in serum, and liver biopsies [17]. In the absence of an established definition, Kamar et al. (2012) defined chronic hepatitis as the carriage of HEV for more than six months [97] and should be diagnosed when HEV replication persists for 3 months after the acute phase [98]. Transplant recipients who developed the chronic state have a significantly shorter time from transplant to diagnosis. As a result, they have lower total lymphocyte counts, CD2, CD3, and CD4 lymphocyte counts [17].

It is suggested that HEV RNA detection always coincides with or follows an ALT increase. Solid organ transplant recipients having chronic HEV infection usually have high levels of liver enzymes and bilirubin [99]. HEV RNA can be detected not only in the serum but also in the cerebrospinal fluid (CSF). Additionally, the viral sequences found in the CSF are reported as clearly distinct from sequences found in the serum [100]. Chronic infection is associated with greater heterogeneity of HEV quasispecies in transplant recipients as compared to patients with resolving infections [20].

Kamar et al. (2011) carried out the largest reported retrospective multicenter study that included 17 centers and 85 transplant patients to assess the relationship between solid organ transplant and HEV infection and to establish the predictive causes for chronic hepatitis. They stated that in solid organ transplant recipients infected with HEV, 60% of cases evolve to chronicity. There is a strong association between usage of tacrolimus, an immunosuppressive drug, and development of chronic hepatitis. Amongst the transplant recipients, 32.1% had HEV clearance after a dose-decrease of immunosuppressive therapy that can be explained by reduced downregulation of T-cell response. Subsequent to HEV clearance, no reappearance of HEV viraemia was reported [101].

### 6.1. Kidney

A study in France estimated HEV seroprevalence in kidney transplant recipients to be 14.5% [17]. Similar studies from different parts showed prevalences of 30.8% (Iran) [102] and 20.5% (India) [103]. Researchers believe that the HEV prevalence rate in transplant recipients is not dependent on its prevalence in the general population [102]. Interestingly, the sera from 205 renal transplant recipients from India, known to be hyperendemic to HEV, tested negative for HEV RNA. Though 20.5% patients did show anti-HEV IgG and 22.4% showed abnormal ALT levels, not a single case of HEV RNA was detected [103]. Previous studies from India have highlighted considerably high rates of HEV infection, as evidenced by reports of seroprevalence [24]. It can thus be reasoned that if *HEV1* infection persists among immunosuppressed persons, at least a few patients with renal transplants would have detectable HEV RNA. Furthermore, the absence of detectable HEV RNA in sera from a large unselected group indicates that risk of chronicity with *HEV1* infection among the immunosuppressed may be low.

HEV infection is reported to cause chronic hepatitis in 14.1% of solid organ transplant recipients in France [104]. *HEV3* was the predominantly genotype reported in HEV infection in HEV-infected solid-organ transplant recipients in France [105], Portugal [106], and Germany [107]. Although *HEV3* is not prevalent in South Africa, a case of *HEV3* infection in a renal transplant recipient was reported [108]. It is therefore possible that the risk of chronicity with *HEV3* infection among immunosuppressed individuals will be high.

Patients with chronic HEV are usually reported with persisting high ALT levels and presence of HEV RNA in their serum [106,109]. However, Gerolami et al. (2008) revealed a kidney transplant patient with chronic HEV and normal serum ALT concentrations. Negative results for seroprevalence despite positive serum HEV RNA in the patient can be because of immunosuppression [110].

Researchers consistently report that chronic HEV infections lead to a rapid cirrhosis, especially in kidney transplantation setting [109,110]. It is therefore of paramount importance to treat chronic HEV infections lest cirrhosis occurs. Therapeutic options for chronic HEV infection in renal transplant recipients is not widely studied and reducing immunosuppressive drugs may be the best possible therapy [109] as a reduction of immunosuppression has shown to cause viral clearance. However, after reducing immunosuppression, patient progress must be monitored closely to circumvent the danger of acute rejection [111]. Interferon-α therapy recommended after other transplants can have adverse effects and high incidence of renal transplant rejection [109].

### 6.2. Liver

Till date, most studies have reported the presence of *HEV3* in liver transplants. Direct molecular testing of liver tissue has also detected HEV-RNA in 1% of the biopsies of the patients [112]. A study in the Netherlands reported the rate of HEV in liver transplant recipients to be 1%. In 2012, a study reported that of 300 liver transplant recipients tested, only one developed chronic HEV infection [99].

Another study on 145 liver transplant recipients with chronic HCV found the seroprevalence of HEV to be 42.5% [113]. Another study reported the rate of chronic hepatitis due to HEV as approximately 60% in solid-organ transplant patients [114]. The conditions of a liver transplant patient may be aggravated by a chronic HEV infection if they have pre-existing liver damage due to HCV. Interestingly the higher rates of HEV seroprevalence in the study involving transplant recipients with chronic HCV can be explained by HCV causing liver injury and HEV being transmitted through blood transfusions. As HEV is a zoonotic pathogen, eating contaminated pork products may cause the chronic infection [101,115,116]. However, reports involving a transplant tourist also suggest that it can result from implantation of an organ from a deceased donor with HEV; the HEV may have reactivated due to immunosuppression [117]. Pegylated interferon-α can cause HEV clearance in liver transplant recipients suffering from chronic active hepatitis [118].

### 6.3. Lung

A recent study comprising 468 lung transplant patients showed that 2.1% of them tested positive for HEV RNA. The patients had elevated liver function test results and the recipients who survived for longer than six months were diagnosed as having chronic HEV infections. All the HEV were of genotype 3 strains, as shown by viral genotyping [119]. Pas et al. (2012) reported chronic hepatitis in 1 out of 53 lung transplant recipients [99].

### 6.4. Heart

Pischke et al. (2012) stated that the HEV prevalence in 274 heart transplant recipients was 11.3% [120]. Ribavirin and not interferon-α is the best therapeutic option as the latter may induce rejections. Ribavirin is effective in clearing HEV infection [121,122], although more dosage studies are warranted; lower doses make the treatment ineffective whereas higher doses may cause anemia.

## 7. Prevention

In endemic countries, HEV spreads via the fecal–oral route and genotypes 1 and 2 are the dominant genotypes. Fecal contamination of drinking water is reported in epidemics [21,24,123]. Direct transmission through person-to-person contact is not a probable cause of HEV infection [124] except in case of vertical transmission from mother to child. Improper water storage and inadequate hand-washing practices, as evidenced by isolation of HEV from hand-rinsed samples, are strongly associated with HEV infections [125]. Living in rural areas is also identified as a risk factor for HEV infection [31]. The spread of HEV infection can be curtailed with use of clean potable water, proper sewage disposal, and preventing contamination of water supplies.

HEV genotypes 3 and 4 are transmitted via animal source food-mediated zoonosis. They predominantly cause disease in middle-aged/elderly males [20]. Consumption of game meats, undercooked boar or offal, swine, pig liver, or raw pig liver sausage are probable risk factors for HEV infection [41,126,127]. Studies speculate people consuming excessive amounts of alcohol have a higher chance of having hepatitis E [128]. Proper cooking of meats and avoiding raw swine products can help reduce HEV infections in industrialized countries.

HEV infection during pregnancy may lead to fulminant hepatic failure and is associated with a high mortality rate that ranges between 20% and 30% [129,130]. These results are of growing concern, especially in India and other endemic countries. The only preventive option is vaccines. Currently, two vaccines are being developed and one is already available in China [7,50]. The first is a recombinant vaccine which was tested in the US at Walter Reed Army Institute of Research; it was found to be safe and immunogenic [131]. Further studies showed that the vaccine effectively prevented hepatitis E. The second vaccine, HEV239, which is now available in China, has had its phase 3 clinical trial in pregnant women [132]. It does not have any negative fetal or maternal outcomes and prevents HEV infections. It can potentially reduce mortality and morbidity caused due to contracting HEV infection in pregnancy.

## 8. Conclusions

In order to prevent the spread of HEV in endemic settings, proper sanitation must be maintained and contamination of water sources must be avoided. *HEV1* and *HEV2* are commonly found in this setting and are transmitted by the fecal–oral route. Educating the masses and exposure to information on HEV and its mode of transmission, along with a strong system of sanitation awareness is key in preventing hepatitis E. *HEV3* and *HEV4* spread can be prevented by adequately cooking swine and game meats. Transplant recipients must be advised to improve personal hygiene, avoid eating high-risk food such as shellfish and pork products, especially pig livers and pig liver sausages. Travel to endemic areas must be limited by transplant patients and well-treated water must only be consumed by them in these areas. Regular testing of transplant recipients on the day of transplant followed by regular yearly testing can discourage chronic HEV infections.

In hemodialysis patients, parenteral route may play a salient role in the transfer of HEV. Hemodialysis is implicated as a source of nosocomial HEV in some studies; however, other researchers report a lack of association between higher HEV prevalence and hemodialysis. In fact, though HCV is associated with higher risk of HEV infections in liver transplant recipients, there is no association between presence of HCV and higher risk of HEV in CHD patients.

HEV seroprevalences in blood donors may shed light on the overall prevalence in a given population. Besides fecal–oral and zoonotic transmission, blood transfusion appears to be a possible mode of HEV transmission. Yearly studies are warranted for each area to prevent widespread dissemination of HEV. However, the HEV antibody detection assays show different results and there is a lack of consensus between them. The same region can be marked as moderate zone of HEV or hyperendemic depending on the assay method used. Seroprevalence data must be interpreted carefully and caution should be applied while comparing seroprevalences from different regions using different assay methods. Additionally, studies must be undertaken to develop standardized detection kits.

There is no standardized treatment for hepatitis E. The best therapeutic option for transplant recipients is ribavirin, interferon, or a combination thereof, depending on the grafted organ. The vaccines developed are based on *HEV1*. Further research must be directed towards the development of HEV vaccines based on immunodominant epitopes of all four human genotypes of HEV.

## Abbreviations

HAV	Hepatitis A Virus
HBV	Hepatitis B Virus
HCV	Hepatitis C Virus
HDV	Hepatitis D Virus
HEV	Hepatitis E Virus
RNA	Ribonucleic acid
FDA	Food and Drug Administration
EIA	Enzyme Immunoassays
RT-PCR	Reverse-transcription polymerase chain reaction
WHO	World Health Organization
IU/ML	International units/milliliter
LAMP	Loop-mediated isothermal amplification
ORF	Open reading frame
IGM	Immunoglobulin M
IGG	Immunoglobulin G
ELISA	Enzyme-linked immunosorbent assay
HIV	Human immunodeficiency Virus
ALT	Alanine aminotransferase
CHD	Chronic hemodialysis
HD	Hemodialysis
CD	Cluster of differentiation
CSF	Cerebrospinal fluid
